# Radiotherapy with or without androgen deprivation therapy in intermediate risk prostate cancer?

**DOI:** 10.1186/s13014-019-1298-9

**Published:** 2019-06-10

**Authors:** Uri Amit, Yaacov R. Lawrence, Ilana Weiss, Zvi Symon

**Affiliations:** 1 Radiation Oncology Department, Chaim Sheba Medical Center, Tel-Hashomer, Israel; 2 The Dr. Pinchas Borenstein Talpiot Medical Leadership Program, Chaim Sheba Medical Center, Tel-Hashomer, Israel; 3 Sackler Faculty of Medicine, Tel Aviv University, Tel Aviv, Israel

## Abstract

**Objective:**

Androgen deprivation therapy (ADT) is beneficial for unfavorable intermediate-risk (IR) prostate cancer patients receiving curative radiotherapy (RT). However, for favorable IR patients the latest NCCN guidelines recommends RT alone. We retrospectively studied treatment patterns and outcomes of patients with IR prostate cancer in our institution over the past two decades.

**Materials and methods:**

Three hundred seventy-three IR prostate cancer patients treated with definitive RT between 5/2002–5/2016 were identified in an institutional review board approved database. All patients received conformal RT to the prostate while the vast majority did not receive nodal radiation. ADT was commenced 2 months prior to RT and was continued for 4 months after RT.

**Results:**

Compared to RT alone, patients receiving combined RT+ ADT had more positive biopsy cores, higher pre-radiation PSA, more IR factors, and were more likely to receive pelvic lymph node radiation. However, there were no differences in failure either biochemical, local or distal, nor on survival between the favorable RT alone and the unfavorable RT+ ADT cohorts, suggesting a beneficial role for ADT. On multivariate analysis, patients 70 years or younger receiving RT alone were at increased risk for biochemical failure during a 6-year follow-up (HR 3.06, *P = 0.025*). Biochemical relapse free survival in patients ≤70 years who received RT alone was 82.1% vs 94.0% for RT + ADT *(P = 0.030)*. There was no difference for combined treatment modality in patients > 70 years *(P = 0.87).*

**Conclusions:**

Men 70 years or younger with favorable IR prostate cancer treated with RT alone to 78 Gy are at increased risk of biochemical failure. Short term ADT should be considered in this cohort of men.

**Electronic supplementary material:**

The online version of this article (10.1186/s13014-019-1298-9) contains supplementary material, which is available to authorized users.

## Introduction

Of the three D’Amico risk stratification groups, intermediate risk (IR) prostate cancer is the most heterogeneous and, therefore, poses a considerable treatment challenge. The current recommendations are based on several randomized controlled clinical trials which included mostly high-risk (HR) patients and tested the potential benefit of adding androgen deprivation therapy (ADT) to conventional radiation therapy (RT) dose, compared with RT alone. The combination of RT+ ADT significantly reduced biochemical failure rates and improved overall- and cancer-specific survival without significantly increasing toxicity compared to RT alone [1–4]. However, it is noteworthy that these trials were performed in the low dose era of up to 70 Gy, in contrast to the standard treatment of 78 Gy commonly prescribed today.

The use of ADT in combination with RT in the IR group has been understudied. D’Amico et al. performed a randomized control trial including 206 patients with clinically localized prostate cancer who were randomized to receive 70 Gy alone or in combination with 6 months of ADT. Although the addition of short-term ADT improved survival, the trial was tainted by the inclusion of HR patients: 5% had a Gleason score of 8–10 and 19.4% had Prostatic Specific Antigen (PSA) 20–40 ng/mL [4]. The DART01/05 GICOR compared the addition of short -versus long-term ADT in combination with modern day, dose escalated RT (76–82) Gy [5]. The study showed that 24 months of ADT combined with higher-dose RT improved biochemical relapse-free survival and overall survival (OS) compared to 4 months in patients with HR prostate cancer while adding no benefit in the IR group. However, all patients received ADT and, therefore, no conclusion can be drawn whether ADT can be omitted altogether. Hence the question remains open whether the addition of ADT is beneficial in IR prostate cancer treated with high-dose RT therapy.

The current National Comprehensive Cancer Network (NCCN) guidelines address this issue by reclassifying IR patients into the favorable risk group who are treated with RT alone and the unfavorable risk group for whom ADT is warranted for patients receiving RT, and considered for patients receiving RT with brachytherapy boost. The intensification of RT with ADT for the unfavorable risk group has been broadly accepted over the past decade. However, the use of ADT in favorable risk patients is still controversial [6–8]. Short term ADT negatively impacts quality of life in sexually active men and may exacerbate co-morbidities such as obesity, diabetes and atherosclerosis [9]. Given the potential side effects of ADT and increased mortality observed in patients with moderate and severe comorbidities [4, 10, 11], there is a need to better define subgroups of patients who may benefit from the combined therapy, while sparring the unwanted health risks associated with ADT in others.

To explore the importance of concomitant ADT in the era of modern RT, we performed a retrospective analysis of patterns of care and outcomes of IR prostate cancer patients treated at our center.

## Patients and methods

### Patients

All IR prostate cancer patients treated with definitive RT therapy at the RT Oncology Department at the Chaim Sheba Medical Center (CSMC) between 5/2002–5/2016 were identified from the hospital’s clinical database following approval of the CSMC ethics committee. IR prostate cancer was defined as having the following characteristics: clinical tumor stage T2b or T2c, Gleason score of 7, or a PSA level of 10–20 ng/mL. Only patients who received 6 months of ADT treatment were included in the RT and ADT group. Patient characteristics, including: age, clinical stage, PSA, Gleason score, number and percentage of biopsy cores involved with tumor, duration of ADT, RT treatment modality, biologic equivalent dose, and Charlson comorbidity index [12] were extracted from the patient’s electronic medical record. Patients were invited for follow-up examination every 6 months to 1 year. Patients who did not attend their routine follow-up visit were contacted by phone and asked to send recent serum PSA tests.

### Planning and treatment guidelines

External beam RT: The prostate and proximal seminal vesicles were contoured on axial images from the treatment planning CT scan, and merged to form the clinical target volume (CTV). The CTV was expanded 1 cm in 3 dimensions, except for 0.7 cm posteriorly to create the planning target volume (PTV). The PTV was planned to 95% of the prescribed dose. Three treatment protocols evolved during the study time as follows: From 2001 to 2009, patients received 3D conformal RT, with a total dose of 78Gy- 82Gy at 2 Gy/fraction (FX); from 2004 to 2011, patients received Intensity-Modulated RT (IMRT) with a total dose of 78Gy- 82Gy at 2 Gy/FX; and from 2010 to 2016, patients received volumetric modulated arc therapy (VMAT) and mild hypo-fractionation to 73.6Gy at 2.3Gy/FX (80Gy 2GyEq., σ/β = 1.5).

Androgen Deprivation Therapy: all patients in the combined RT+ ADT group received two depot subcutaneous injections of Goserelin 10.8 mg every 3 months to a minimum of 6 months effect. Bicalutamide 50 mg was co-administered in the first month to prevent testosterone flare. Patients in the RT alone group did not receive any hormonal therapy during RT.

### Statistics

Statistical analysis was performed using SPSS version 25 (SPSS, Inc., Chicago, Illinois). Continuous variables are expressed as mean ± SD and categorical variables as percentages. Comparisons of variables were performed using a two-tailed Student’s t-test for continuous variables, and Chi-square for categorical variables. To identify parameters associated with biochemical failure, a univariate Cox proportional hazard analysis was performed. The parameters with *P* values *< 0.1* thus identified were selected for consideration and entered simultaneously in a multivariate Cox proportional hazard analysis. To depict the occurrence of local failure, biochemical failure, distant metastasis, restarting ADT and mortality during follow-up, taking into account the censored data, Kaplan-Meier method was employed. Survival differences were evaluated using the log-rank test. For all calculations *P* values *< 0.05* were considered statistically significant.

## Results

### Study population and characteristics

During the study period, 373 patients diagnosed with IR prostate cancer were treated in the Radiation Oncology Department of the CSMC; 196 patients (52.5%) were treated with RT with concomitant short-term (6 months) ADT, and 177 patients (47.5%) with RT alone. Table 1 shows population and treatment-related characteristics of patients treated with RT and short-term ADT and RT alone. Patients in both arms were similar in terms of mean age at time of therapy, Gleason score at biopsy, fiducials implants, RT treatment modality (3D conformal RT, IMRT, VMAT), and the Charlson comorbidity score. The mean follow-up time was 55.9 ± 37.2 months and did not significantly differed between the two treatment groups. However, compared to patients treated with RT alone, patients treated with a combination of RT+ ADT had significantly more unfavorable risk factors including: higher PSA levels before RT treatment, more intermediate-risk risk factors, and a higher percent of positive cores on biopsy. Moreover, patients receiving RT and ADT had higher T-stage, however the difference did not reach statistical significance (*P = 0.074*). The vast majority of patients in both groups did not receive pelvic lymph node RT (93%), and a higher fraction of patients receiving RT and ADT were administered pelvic lymph node RT.

**Table 1:** Patient characteristics and treatment

Characteristic	IR RT alone (*n* = 177)	IR RT+ ADT (*n* = 196)	*P*
Age (mean ± SD, years)	72 ± 5.34	72 ± 5.85	0.934
Pretreatment PSA (ng/mL)	7.88 ± 3.55	9.34 ± 3.97	0.001>
Fiducials implant % (n)	71.8 (127)	65.3 (128)	0.181
Gleason score % (n)			0.257
5 or 6	19.8 (35)	15.8 (31)	
7 (3 + 4)	52.5 (93)	49.0 (96)	
7 (4 + 3)	27.7 (49)	35.2 (69)	
T- stage % (n)			0.074
T1	31.4 (50)	23.4 (37)	
T2a-b	64.8 (103)	67.7 (107)	
T2c	3.8 (6)	8.9 (14)	
No. IR risk factors % (n)			0.001>
1	65.0 (115)	42.6 (83)	
2	31.1 (55)	48.7 (95)	
3	4.0 (7)	8.7 (17)	
RT treatment modality % (n)			0.929
3D	17.2 (29)	15.7 (29)	
IMRT	23.1 (39)	23.8 (44)	
VMAT	59.8 (101)	60.5 (112)	
RT lymph-nodes % (n)	2.8 (5)	10.7 (21)	0.003
Positive biopsy cores % (mean ± SD)	45.33 ± 25.56	52.56 ± 27.61	0.017
Charlson comorbidity index			0.774
2	49.4 (80)	52.0 (93)	
3	16.7 (27)	14.0 (25)	
4≤	34.0 (55)	34.1 (61)	
Months follow-up	57.6 ± 36.9	54.3 ± 37.4	0.383

### Patterns of treatment failure and outcome

**Fig. 1:**
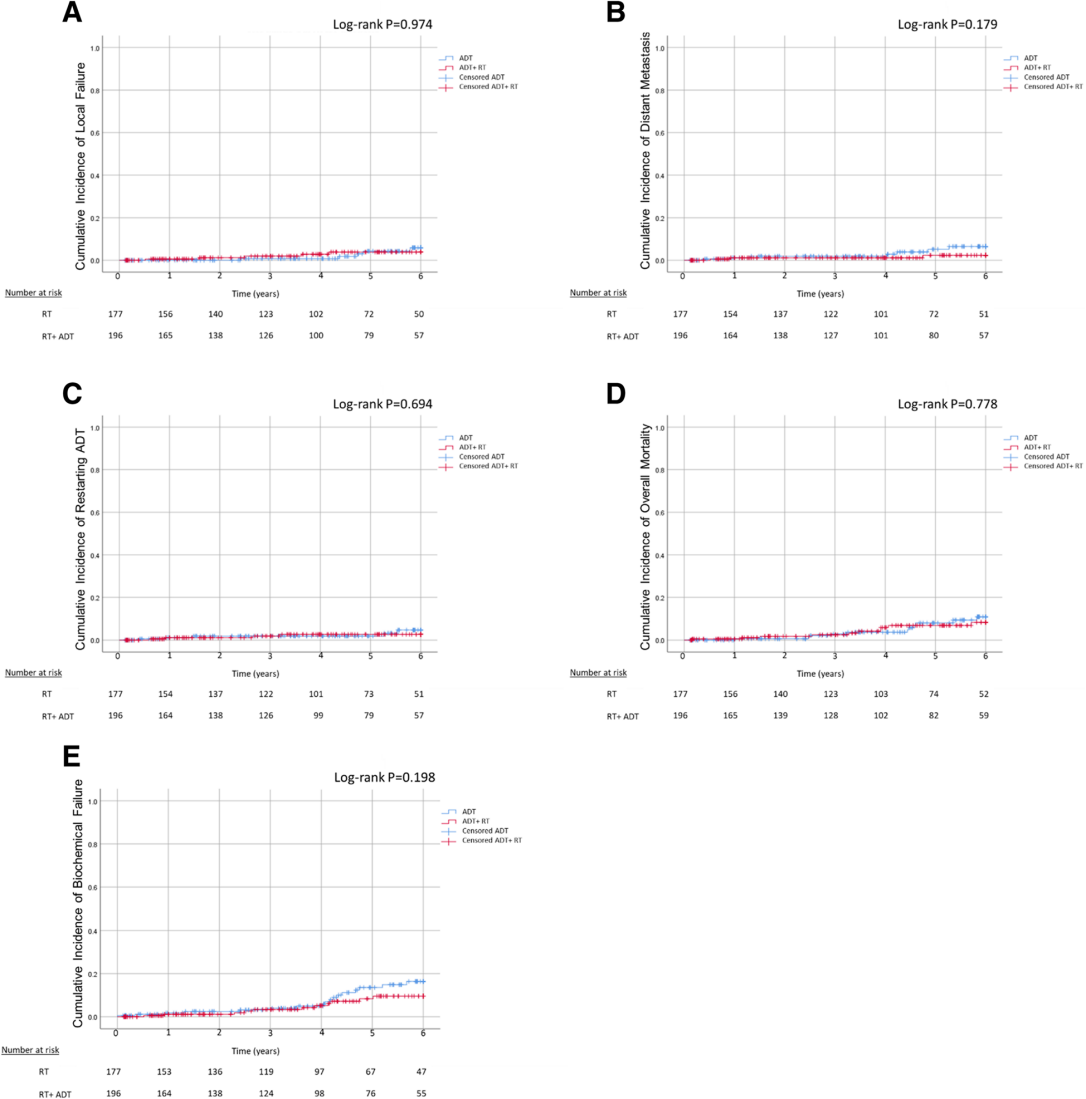
Patterns of failure in IR prostate cancer patients receiving RT alone or combined RT and ADT, during the 6-year follow-up. **a** Local failure **b** Distant metastasis **c** Restarting ADT **d**. Overall mortality **e**. Biochemical failure

Of the 373 patients included in the study, 28 had biochemical failure, as defined by the Phoenix criteria [13], 10 had local recurrence in the pelvis, 10 had distal metastatic disease, and 9 patients began or restarted ADT treatment during the 6-year follow-up. During that period, 21 of the 373 patients died, with no difference in overall survival between the two treatment groups. None of the patients died from prostate cancer-specific mortality during the study period. Figure 1 compares patterns of failure between IR prostate cancer patients treated with RT and RT+ ADT during the 6-year follow-up. No significant difference in treatment failure (biochemical, local, distal or retreatment with ADT) was demonstrated between patients treated with RT+ ADT compared to RT alone.

### Predictors of biochemical failure in patients treated with RT alone

We identified subgroups of patients who were treated with RT alone who were at increased risk for biochemical failure. Table 2 shows the results of a univariate Cox proportional hazard analysis using patient age (age ≤ 70 years vs. age > 70 years), pretreatment PSA level, Gleason score (5 or 6 vs. 7 (3 + 4) vs. 7 (4 + 3)), T-stage (T1 to T2a vs. T2b to T2c), cumulative number of IR factors (1 vs. 2 vs. 3), % positive cores at biopsy, and Charlson comorbidity index (2 vs. 3 vs. ≥4). Parameters that reached a *P < 0.1* level of statistical significance in the univariate analysis were selected for inclusion in a multivariate Cox proportional hazard analysis. Only age ≤ 70 years was found to be a significant predictor of biochemical failure in IR prostate cancer patients treated with RT alone, with a hazard ratio of 3.06. No significant differences in the occurrence of local failure, distal failure or mortality was noted between patients over and under 70 years treated with RT (Additional file 1: Figure S1).

**Table 2:** Univariate and multivariate Cox regression analysis for 6-year biochemical failure in IR patients treated with RT alone

Characteristic	Covariate type	Number at risk	Cumulative probability of biochemical failure	Univariate analysis				Multivariate analysis			
				HR	P	95% C.I.		HR	P	95% C.I.	
						Lower	Upper			Lower	Upper
Age ≤ 70 years	Categorical	56	17.9%	3.32	0.018	1.226	8.464	3.06	0.025	1.153	8.138
Pretreatment PSA (ng/mL)	Continuous	177		1.03	0.684	0.899	1.176				
Gleason score	Categorical	177									
5 or 6		35	5.7%	1	reference						
7 (3 + 4)		93	6.5%	1.16	0.852	0.235	5.772	1.42	0.667	0.285	7.128
7 (4 + 3)		49	18.4%	3.16	0.141	0.685	14.656	3.32	0.125	0.717	15.383
T- stage T2b to T2c	Categorical	61	11.5%	1.73	0.280	0.641	4.664				
No. IR risk factors	Categorical	177									
1		115	8.7%	1	reference						
2		55	10.9%	1.76	0.278	0.635	4.856				
3		7	14.3%	2.87	0.319	0.361	22.750				
Positive biopsy cores (%)	Continuous	154		1	0.917	0.981	1.022				
Charlson comorbidity index	Categorical	162									
2		69	11.6%	1	reference						
3		27	0%	0.00	0.977	0.000	0.000				
≥4		49	10.9%	0.72	0.836	0.309	2.261				

**Fig. 2:**
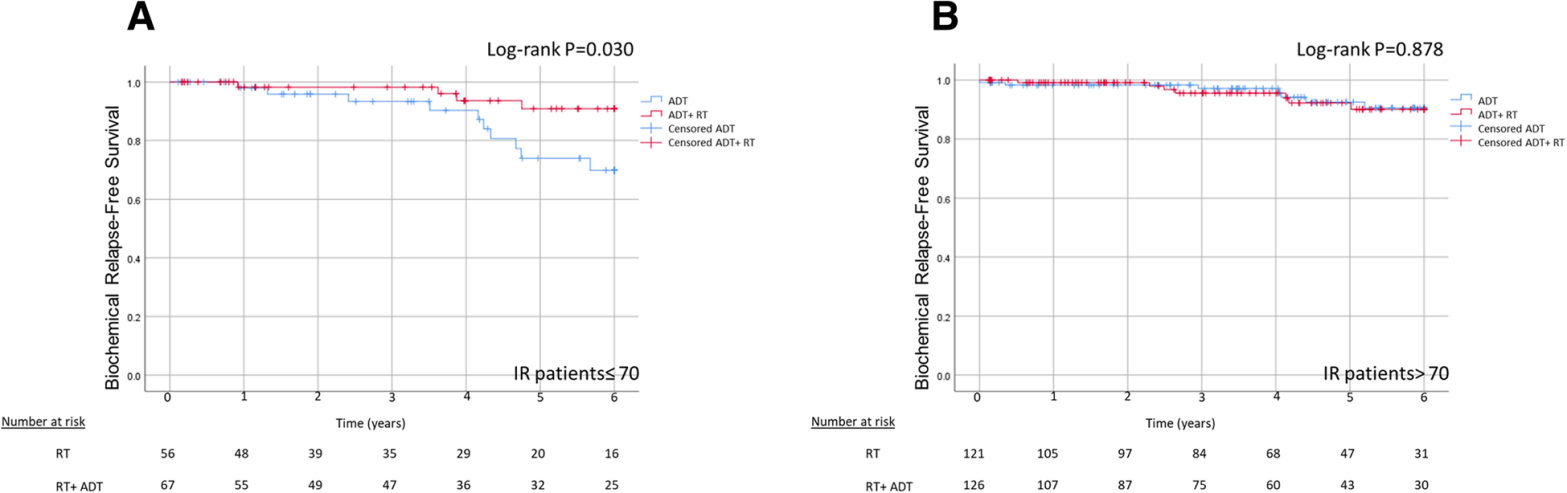
Six years biochemical relapse-free survival in IR prostate cancer treated with RT alone or combined RT and ADT **a** Patients ≤70 years old **b** Patients > 70 years old

We then tested the effect of age below and above 70 years in the RT alone cohort versus the RT+ ADT cohort. Age 70 was chosen as a cut-off because it is widely used for treatment related decision making in localized prostate cancer. Radical prostatectomy is preferred in men younger than 70 years, whereas radiation therapy is applied predominantly in patients older than 70 years [14]. Figure 2a Shows significantly inferior biochemical relapse free survival (BRFS) in men ≤70 years treated with RT alone versus RT+ ADT (82.1 vs. 94% at 6 years, *P = 0.030*). However, in men age > 70 years there was no difference between RT alone and the RT+ ADT (94.4% vs. 94.2%, *P = 0.878*, Fig. 2b).

## Discussion

In this study we compared IR prostate cancer patients treated with 78 Gy RT with and without the addition of ADT. Patients treated with RT+ ADT comprised a predominantly unfavorable risk group with higher PSA levels, more cumulative IR risk factors, a higher percent of positive cores on biopsy, and a quasi-significant higher T-stage. Despite these unfavorable features, the clinical outcome of both groups was entirely comparable, suggesting that addition of ADT to RT improves outcomes of the unfavorable risk group making them indistinguishable from outcomes of favorable risk group patients treated with RT alone.

**Fig. 3:**
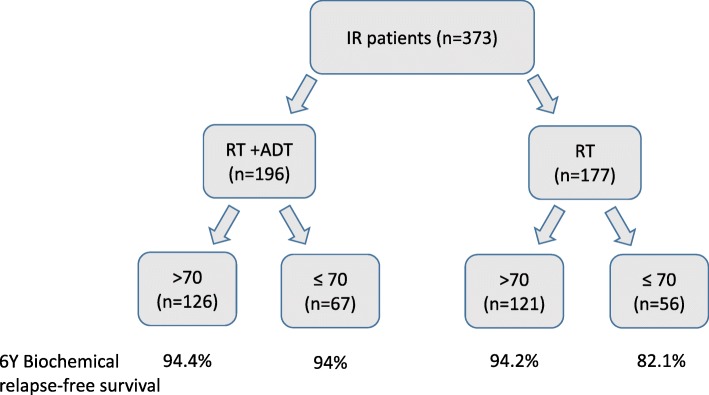
Study summary scheme

However, on multivariate Cox regression analysis of patients with favorable characteristics receiving RT alone, age emerged as a highly significant predictor. Patients 70 years or younger were at increased risk for biochemical failure during a 6-year follow up. This was not the case for men older than 70 years (Fig. 3).

The impact of age on outcomes of prostate cancer treated with RT has not been extensively reported. Studies examining the association between age at diagnosis of prostate cancer and risk for biochemical failure after radiation show conflicting results. In accordance with our results, Rosser et al. reviewed the medical records of 964 patients who received definitive radiotherapy as the only treatment, at the M. D. Anderson Cancer Center. With a median follow-up of 48 months, they showed that men with prostate cancer aged ≤60 years, have a higher rate of biochemical failure at 5 and 7 years (45% vs. 35% for 5 years, *P < 0.05* and 53% vs. 41% for 7 years, *P < 0.05*) [15]. However, these results were challenged by Zelefsky et al. who found no difference in biochemical free survival for men younger than 60 receiving definitive dose escalated prostate RT [16]. Klayton et al. also examined the impact of younger age on biochemical failure following RT. In their study the 8-year incidence of biochemical failure in men aged 60 and younger was 27.3%, compared to 23.3% in the older patients *(P = 0.29).* Although this difference is not statistically significant, it is noteworthy that the younger age group had a significantly less aggressive disease with 40% of the patients categorized as low-risk compared to only 29% of the patients over 60 [17].

We are aware of several potential limitations in our study. First, this investigation is based on a single center experience, with a relatively small patient population and relatively low number of biochemical failures. Dose escalation was moderate, primarily 78 Gy, and superior results have been reported with more intense dose escalation such as utilized with brachytherapy boosts or Stereotactic Body RT [18]. Mean follow-up was limited to four and a half years, however this was enough to detect more failures in younger men. Furthermore, due to the retrospective nature of the study, an inadvertent selection bias cannot be excluded. Selection bias may account for higher rates of genetic predisposition and inherited mutations in the younger age group leading to a more aggressive disease not identifiable in the current prostate risk groups. Last, a small yet significant difference in pelvic nodal RT was noticed between the RT and RT+ ADT groups. Although most patients in both groups did not receive radiation, this could have reduced the observed differences in outcomes between the two patients’ groups. Despite these caveats, this study has identified a population of younger men with IR prostate cancer who are at greater risk for biochemical failure when treated with RT alone.

## Conclusion

IR prostate cancer patients have recently been classified as favorable and unfavorable in order to select RT as monotherapy or to combine RT with short term ADT. This study represents a cohort of patients treated according to this paradigm and found that men 70 years or younger with favorable IR prostate cancer treated with RT alone are at increased risk of biochemical failure. These findings suggest that age may be considered a risk factor when stratifying men with IR prostate cancer for primary RT to standard 78 Gy doses. Short term ADT should be co-administered to dose escalated RT in this cohort of men.

## Additional file

Additional file 1: **Figure S1.** Patterns of failure of IR prostate cancer patients under and over 70 years, receiving RT alone during 6-year follow-up. A. Local failure B. Distant metastasis C. Overall mortality (DOCX 176 kb)

## Abbreviations

ADT: Androgen deprivation therapy
CSMC: Chaim Sheba Medical Center
CTV: Clinical target volume
FX: Fraction
IMRT: Intensity-Modulated RT
IR: Intermediate-risk
NCCN: National Comprehensive Cancer Network
OS: Overall survival
PSA: Prostatic Specific Antigen
PTV: Planning target volume
RT: Radiotherapy
