# Supplementary material for: Radiotherapy with or without androgen deprivation therapy in intermediate risk prostate cancer?
Source: Radiat Oncol. 2019 Jun 10;14:99. doi: 10.1186/s13014-019-1298-9 (PMC6558831; doi:10.1186/s13014-019-1298-9)
Supplement: Supplementary file 1 — Figure S1. Patterns of failure of IR prostate cancer patients under and over 70 years, receiving RT alone during 6-year follow-up. A. Local failure B. Distant metastasis C. Overall mortality (DOCX 176 kb) [file 13014_2019_1298_MOESM1_ESM.docx]

Additional file 1

Figure S1. Patterns of failure of IR prostate cancer patients under and over 70 years, receiving RT alone during 6-year follow-up. A. Local failure B. Distant metastasis C. Overall mortality.

| 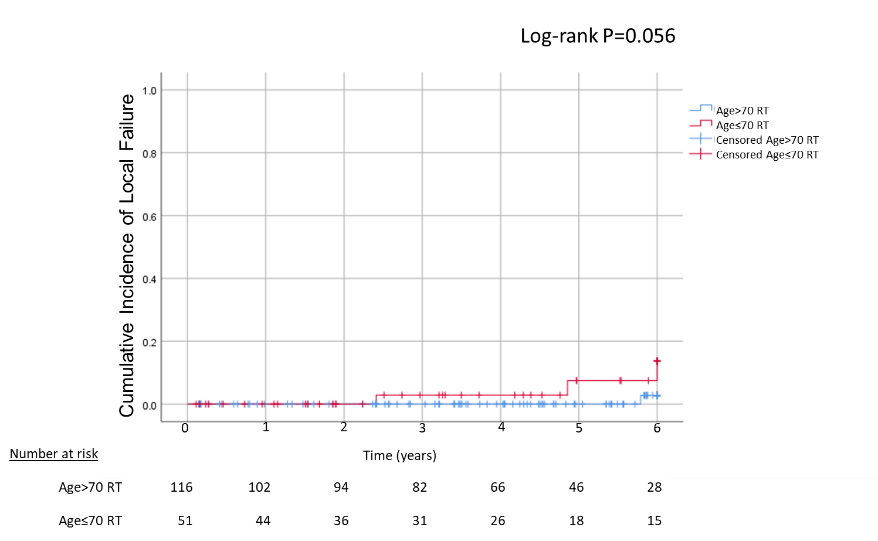  A. | 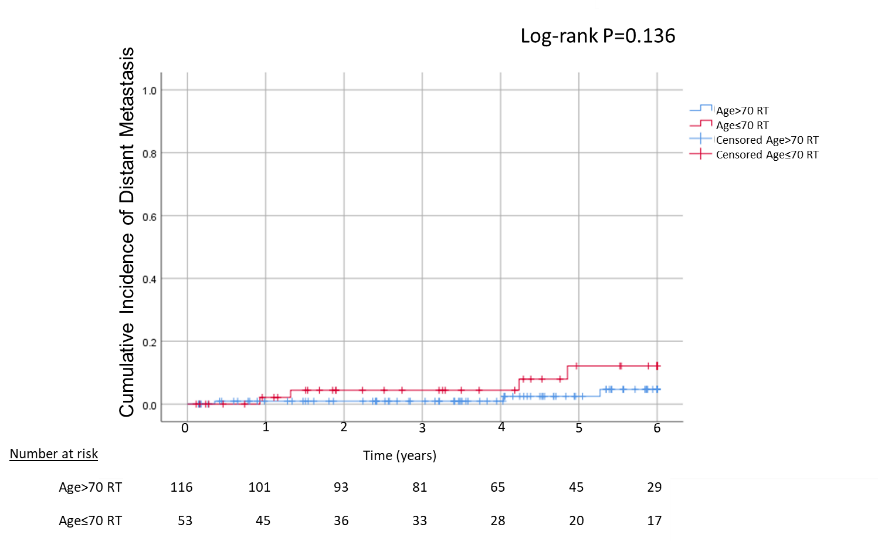  B. |
| --- | --- |
| 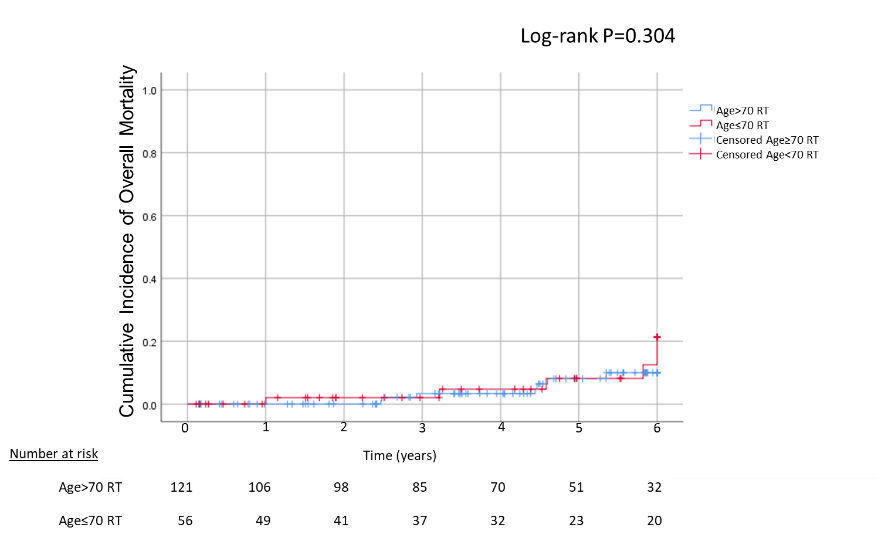  C. |  |
